# Supplementary material for: Temporal structure of mouse courtship vocalizations facilitates syllable labeling
Source: Commun Biol. 2020 Jun 26;3:333. doi: 10.1038/s42003-020-1053-7 (PMC7320152; doi:10.1038/s42003-020-1053-7)
Supplement: Supplementary file 1 — Supplementary Information [file 42003_2020_1053_MOESM1_ESM.pdf]

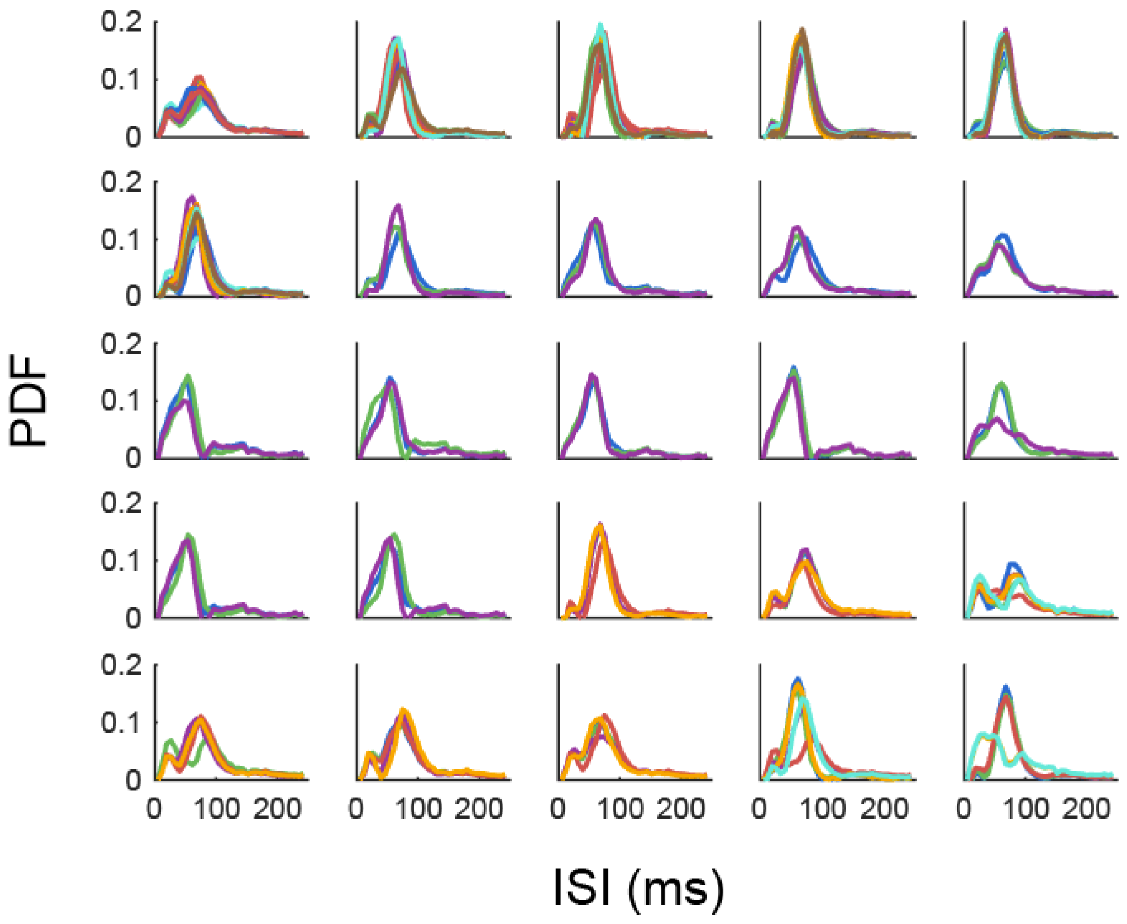

SUPPLEMENTARY FIGURE 1 | ISI distributions of individual mice. A matrix of 5x5 ISI distributions, where each cell corresponds to an individual mouse and each colored line represents a single session. Some of the mice have stereotypic bi-modal distributions (peaks around 20 ms and 70 ms) while others have a single peak around 60 ms.

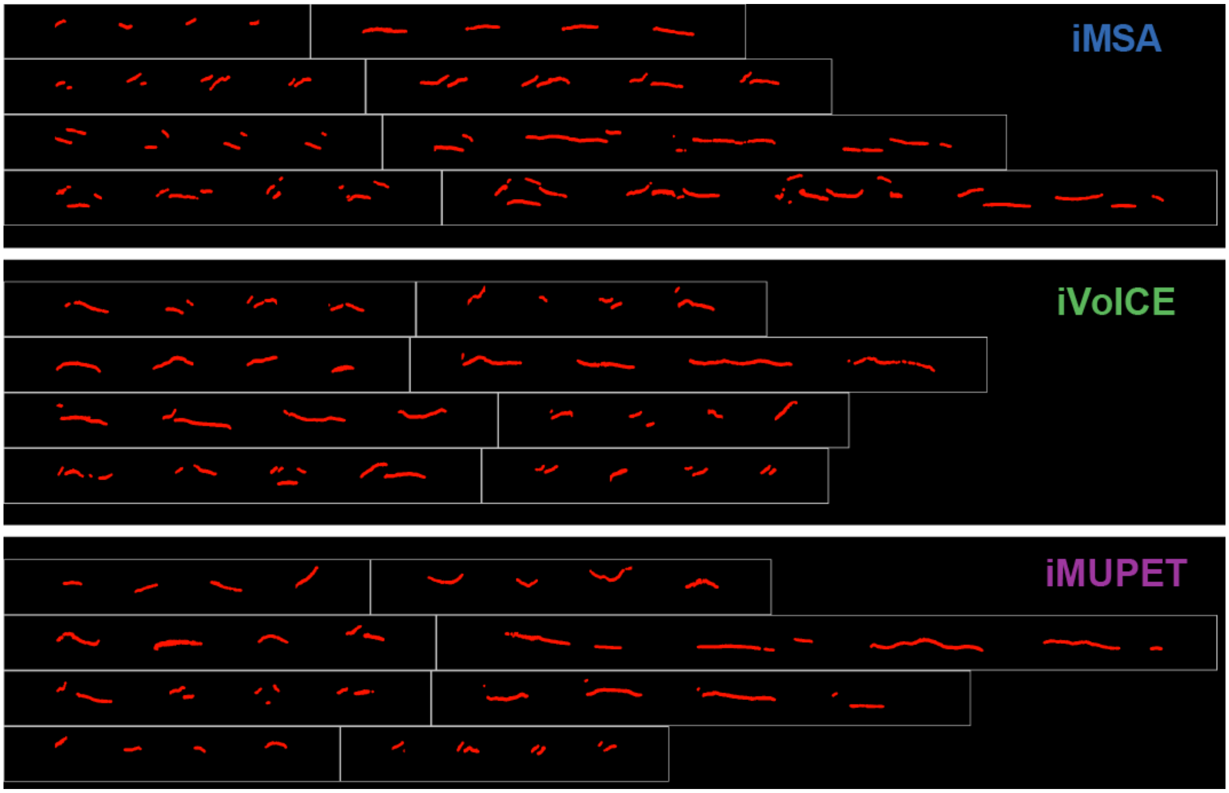

SUPPLEMENTARY FIGURE 2 | Example syllables with one of eight different labels assigned by each algorithm.
Four example syllables are presented for each one of the eight labels that were created by the labeling algorithms.

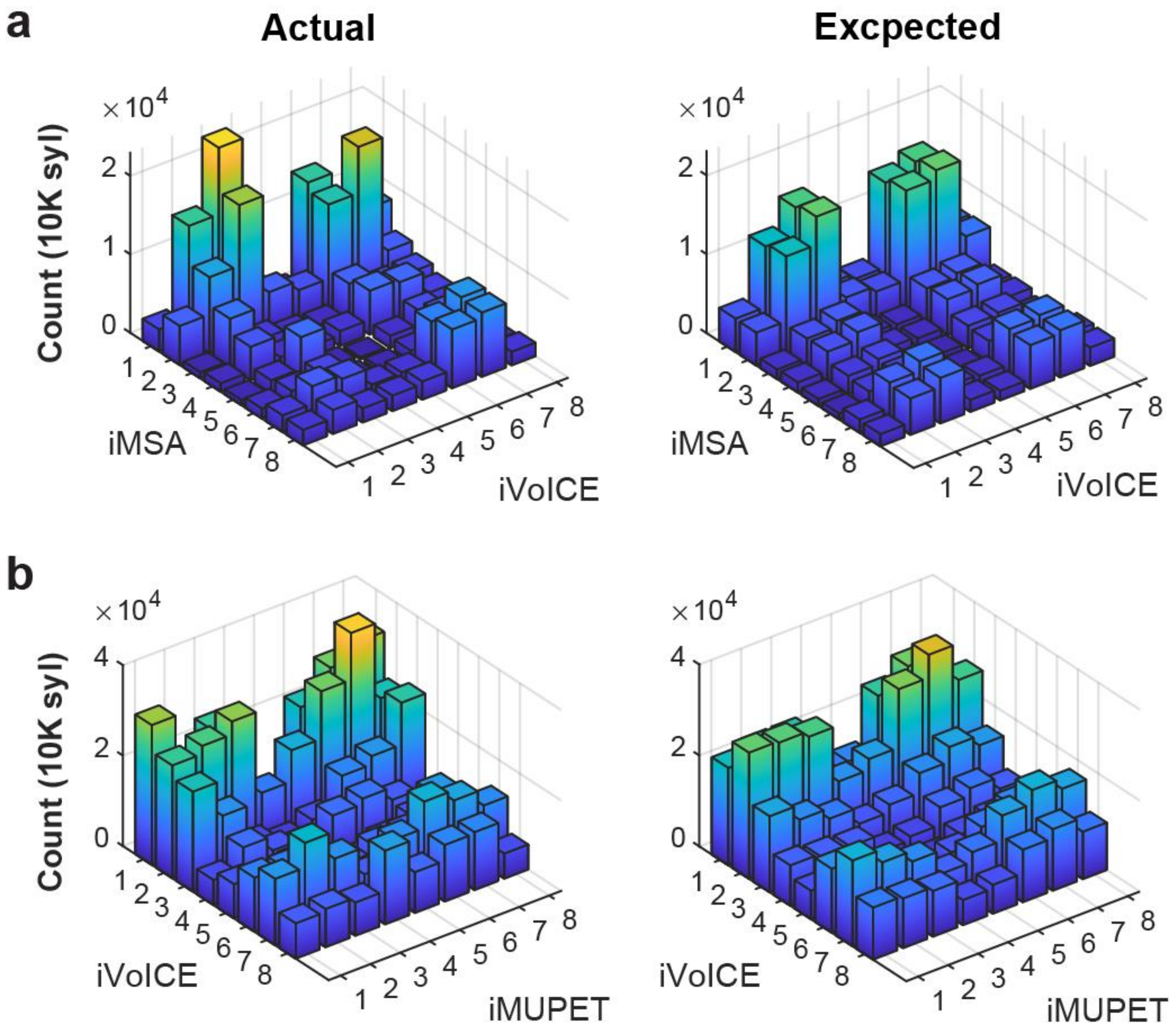

SUPPLEMENTARY FIGURE 3 | Comparison between joint distribution and expected joint distribution of assigned labels by the different algorithms.

a Joint distribution of the lables of iMSA and iVoICE for all USVs in the dataset and the expected joint distribution assuming independence.

b Same comparison, this time between IVoICE and iMUPET.

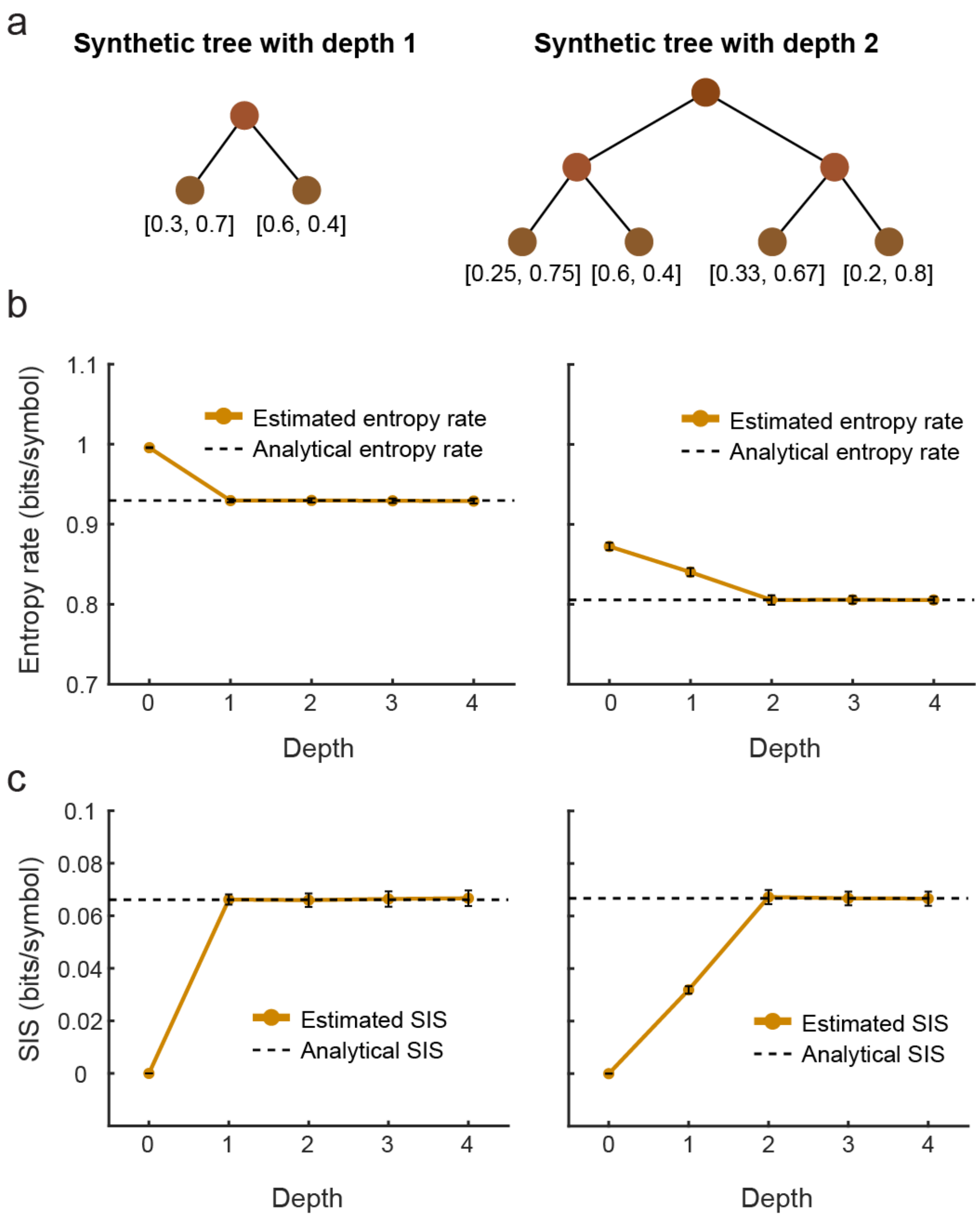

SUPPLEMENTARY FIGURE 4 | Comparison between analytical and estimated entropy rate and SIS values of synthetic Markov models of various orders

a Visualization of two different synthetic Markov models of orders 1 and 2. The leaves of the trees hold the transition probabilities.

b Analytical calculation of the entropy rates of each model is presented with a dashed black line. Estimates of the entropy rate for 25 repetitions (mean +- 2 s.d.) are represented in brown. In each repetition, a sequence composed of 120,00 syllables (equivalent to the amount of data used to calculate the values in figures 4, 5 and 7) is generated from the tree based on the transition probabilities. Then, an estimated tree is built from the generated sequences. Finally, the estimated entropy rate is calculated from the tree.

c Analytical calculation of the SIS of each model as a dashed black line. Estimated values of the SIS, using the same process as B, as a solid brown line.

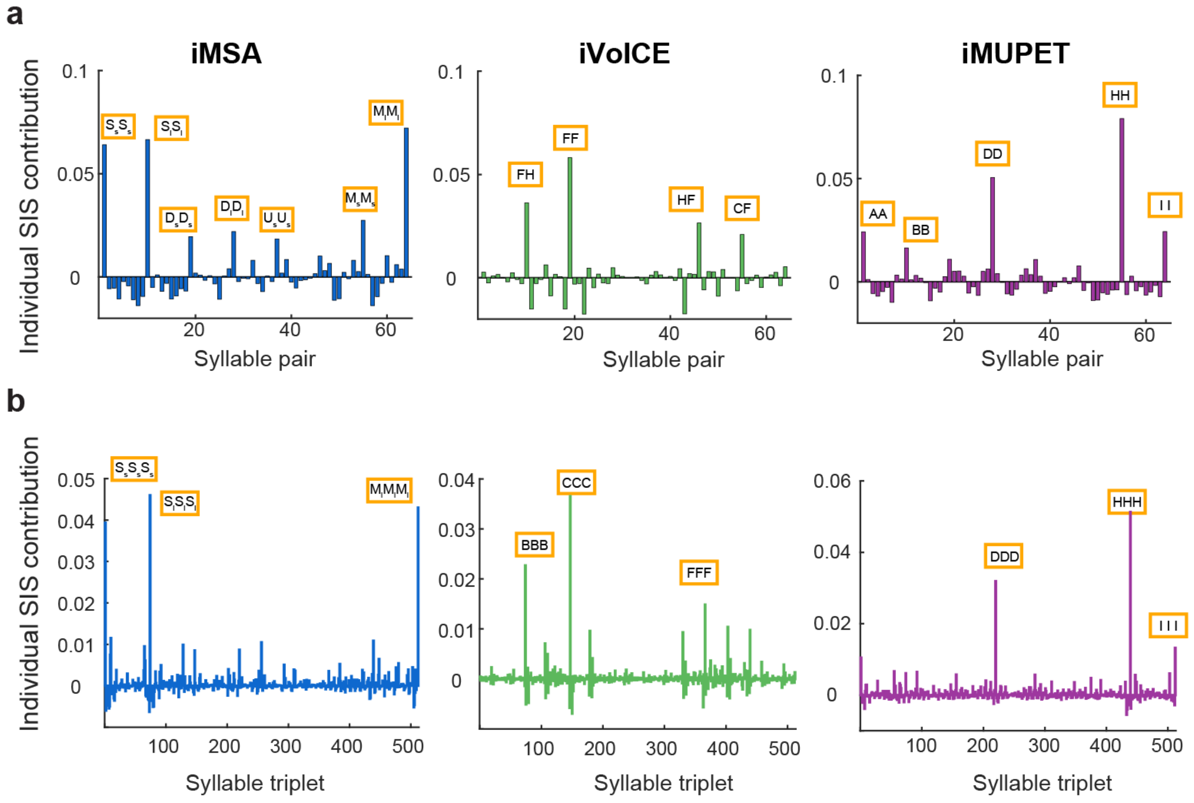

SUPPLEMENTARY FIGURE 5 | SIS contribution of individual pairs and triplets.

a The contribution of each pair to the total SIS value (depth 1).

b The contribution of each triplet to the total SIS values (depth 2).

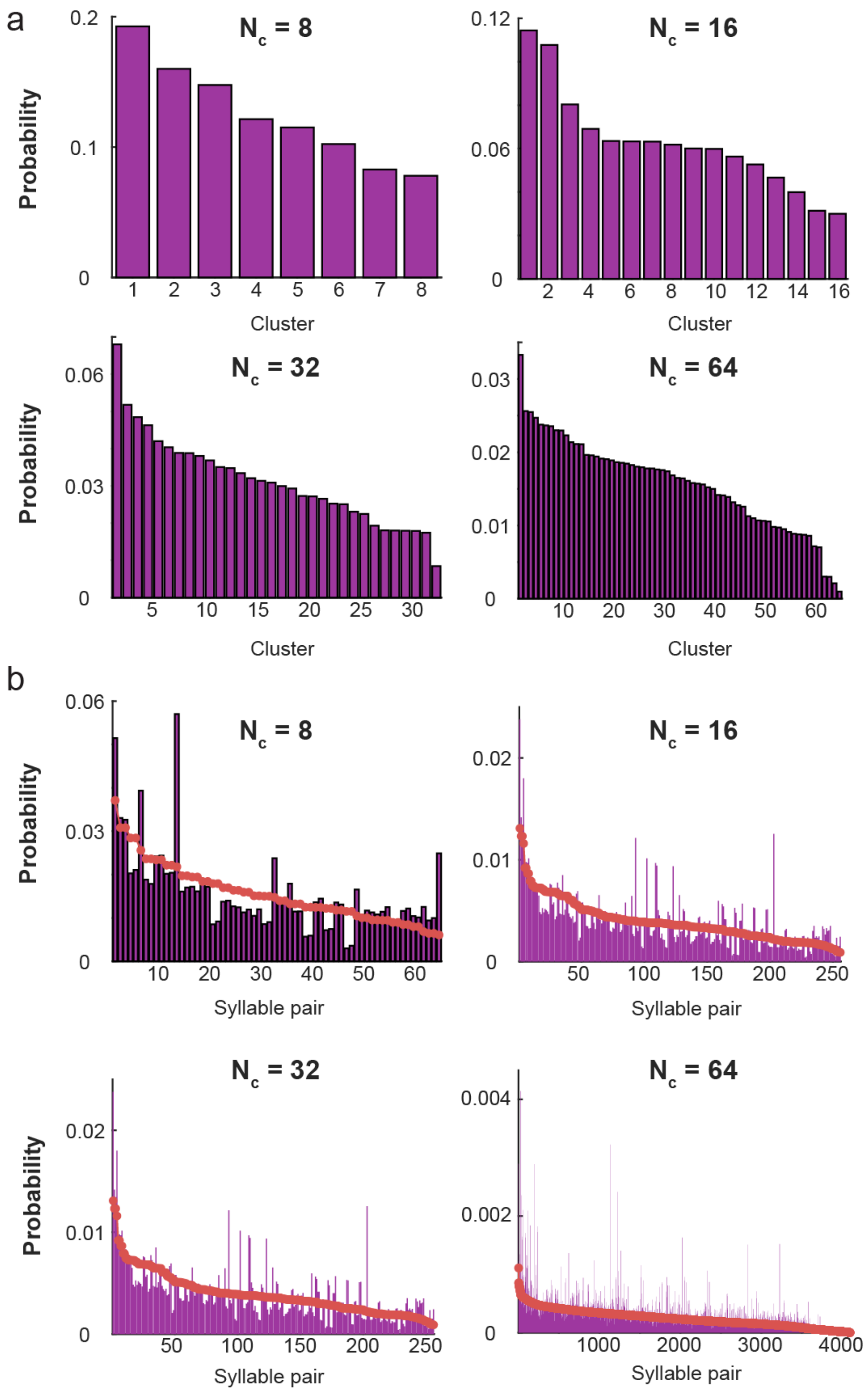

SUPPLEMENTARY FIGURE 6 | Distributions of syllables and pairs after applying iMUPET with an increasing N_c.

a The 0th-order distributions of the labeled USVs from the database are shown for iMUPET with increasing N_c.

b The distributions for pairs of USVs are shown for each N_c. The red line depicts the predicted distributions derived from A assuming independence. The histograms are sorted by the expected distribution.

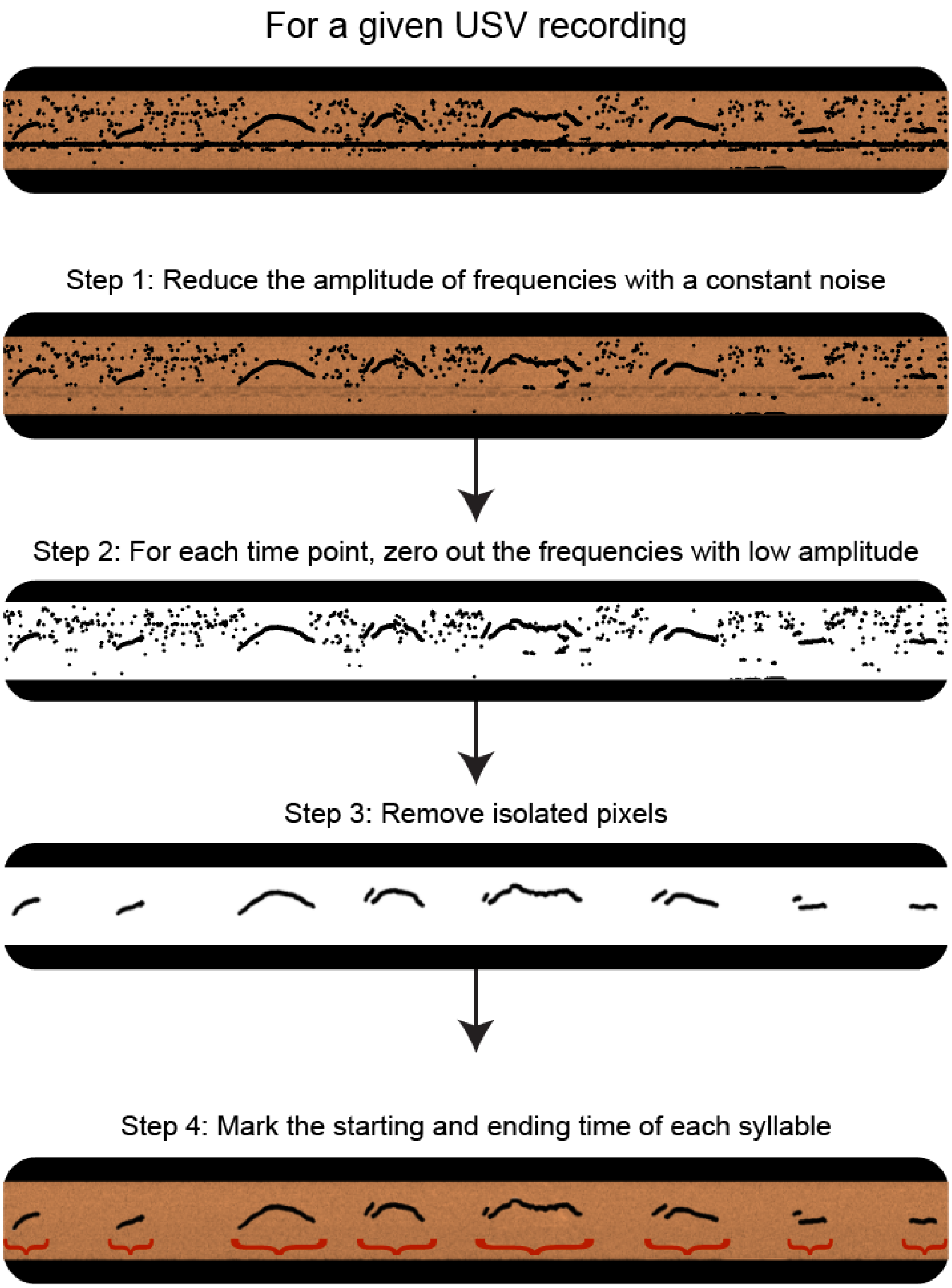

SUPPLEMENTARY FIGURE 7 | An illustration of the main phases of the parsing algorithm. The algorithm converts the USV recording to a spectrogram and applies three main stages of noise removal (steps 1-3). Then, the algorithm scans the clean spectrogram, detects the starting and ending time of each syllable and returns them as a list (step 4).
Step 1: Some of the recordings contain one or more frequencies with a constant noise. The first step of the algorithm searches for this type of noise and reduces the amplitude of the corresponding frequencies.
Step 2: For each time point, frequencies with low amplitudes are set to zero.
Step 3: Pixels that the sum of amplitudes of their neighbors is low are set to zero.
Step 4: The starting and ending point of each syllable is detected.

File name	Mouse ID	Strain	Session	Total sessions	Recording length (sec)	Syllable rate (syl/sec)
USV_HOS_M101_S1.wav	101	C57BL/6	1	13	602.087712	1.748914617
USV_HOS_M101_S2.wav	101	C57BL/6	2	13	601.170208	0.612139449
USV_HOS_M101_S3.wav	101	C57BL/6	3	13	601.399584	0.580313005
USV_HOS_M101_S4.wav	101	C57BL/6	4	13	600.514848	0.316395174
USV_HOS_M101_S5.wav	101	C57BL/6	5	13	605.298976	0.251115574
USV_HOS_M101_S6.wav	101	C57BL/6	6	13	603.136288	0.950034033
USV_HOS_M101_S7.wav	101	C57BL/6	7	13	617.718048	0.048565847
USV_HOS_M101_S8.wav	101	C57BL/6	8	13	601.13744	0.545632293
USV_HOS_M101_S9.wav	101	C57BL/6	9	13	599.957792	0.626710754
USV_HOS_M101_S10.wav	101	C57BL/6	10	13	602.710304	0.645417869
USV_HOS_M101_S11.wav	101	C57BL/6	11	13	600.056096	0.679936431
USV_HOS_M101_S12.wav	101	C57BL/6	12	13	604.381472	1.103607624
USV_HOS_M101_S13.wav	101	C57BL/6	13	13	601.268512	0.949658911
USV_HOS_M102_S1.wav	102	C57BL/6	1	14	601.563424	2.724567243
USV_HOS_M102_S2.wav	102	C57BL/6	2	14	606.249248	1.108454983
USV_HOS_M102_S3.wav	102	C57BL/6	3	14	601.13744	0.971491644
USV_HOS_M102_S4.wav	102	C57BL/6	4	14	600.547616	0.624430087
USV_HOS_M102_S5.wav	102	C57BL/6	5	14	601.399584	0.21449965
USV_HOS_M102_S6.wav	102	C57BL/6	6	14	599.957792	0.976735377
USV_HOS_M102_S7.wav	102	C57BL/6	7	14	600.1544	0.50487008
USV_HOS_M102_S8.wav	102	C57BL/6	8	14	600.31824	0.53138482
USV_HOS_M102_S9.wav	102	C57BL/6	9	14	609.919264	1.498558996
USV_HOS_M102_S10.wav	102	C57BL/6	10	14	675.127584	1.610066046
USV_HOS_M102_S11.wav	102	C57BL/6	11	14	600.580384	0.809217239
USV_HOS_M102_S12.wav	102	C57BL/6	12	14	604.41424	0.964570259
USV_HOS_M102_S13.wav	102	C57BL/6	13	14	600.31824	2.988414945
USV_HOS_M102_S14.wav	102	C57BL/6	14	14	600.252704	0.961261809
USV_HOS_M103_S1.wav	103	C57BL/6	1	14	600.383776	2.218580937
USV_HOS_M103_S2.wav	103	C57BL/6	2	14	604.610848	0.360562502
USV_HOS_M103_S3.wav	103	C57BL/6	3	14	606.21648	0.552607874
USV_HOS_M103_S4.wav	103	C57BL/6	4	14	600.351008	0.374780748
USV_HOS_M103_S5.wav	103	C57BL/6	5	14	599.957792	0.465032714
USV_HOS_M103_S6.wav	103	C57BL/6	6	14	603.75888	0.740361782
USV_HOS_M103_S7.wav	103	C57BL/6	7	14	600.056096	0.338301704
USV_HOS_M103_S8.wav	103	C57BL/6	8	14	600.187168	0.376549203
USV_HOS_M103_S9.wav	103	C57BL/6	9	14	620.30672	0.835070753
USV_HOS_M103_S10.wav	103	C57BL/6	10	14	598.941984	1.9183828
USV_HOS_M103_S11.wav	103	C57BL/6	11	14	600.80976	0.925417723
USV_HOS_M103_S12.wav	103	C57BL/6	12	14	600.449312	1.687069965
USV_HOS_M103_S13.wav	103	C57BL/6	13	14	602.054944	1.242411523
USV_HOS_M103_S14.wav	103	C57BL/6	14	14	610.935072	0.554887116
USV_HOS_M104_S1.wav	104	C57BL/6	1	14	600.64592	2.159342063

USV_HOS_M104_S2.wav	104	C57BL/6	2	14	601.563424	0.335791692
USV_HOS_M104_S3.wav	104	C57BL/6	3	14	604.41424	0.494693838
USV_HOS_M104_S4.wav	104	C57BL/6	4	14	600.842528	0.296250668
USV_HOS_M104_S5.wav	104	C57BL/6	5	14	600.908064	1.080032103
USV_HOS_M104_S6.wav	104	C57BL/6	6	14	602.022176	2.199254533
USV_HOS_M104_S7.wav	104	C57BL/6	7	14	590.16016	0.549003511
USV_HOS_M104_S8.wav	104	C57BL/6	8	14	600.383776	0.534658019
USV_HOS_M104_S9.wav	104	C57BL/6	9	14	597.303584	1.349397562
USV_HOS_M104_S10.wav	104	C57BL/6	10	14	600.088864	1.899718638
USV_HOS_M104_S11.wav	104	C57BL/6	11	14	600.187168	0.891388601
USV_HOS_M104_S12.wav	104	C57BL/6	12	14	601.7928	1.211380395
USV_HOS_M104_S13.wav	104	C57BL/6	13	14	600.80976	0.669096987
USV_HOS_M104_S14.wav	104	C57BL/6	14	14	611.819808	0.286031929
USV_HOS_M105_S1.wav	105	C57BL/6	1	14	512.401696	2.257994088
USV_HOS_M105_S2.wav	105	C57BL/6	2	14	601.825568	0.618119302
USV_HOS_M105_S3.wav	105	C57BL/6	3	14	629.088544	0.370377115
USV_HOS_M105_S4.wav	105	C57BL/6	4	14	599.99056	0.196669761
USV_HOS_M105_S5.wav	105	C57BL/6	5	14	600.187168	0.343226265
USV_HOS_M105_S6.wav	105	C57BL/6	6	14	600.940832	1.153191734
USV_HOS_M105_S7.wav	105	C57BL/6	7	14	580.460832	0.592632579
USV_HOS_M105_S8.wav	105	C57BL/6	8	14	600.711456	0.489419666
USV_HOS_M105_S9.wav	105	C57BL/6	9	14	607.297824	0.65206886
USV_HOS_M105_S10.wav	105	C57BL/6	10	14	611.393824	1.341197716
USV_HOS_M105_S11.wav	105	C57BL/6	11	14	611.557664	1.057954201
USV_HOS_M105_S12.wav	105	C57BL/6	12	14	573.546784	0.188301989
USV_HOS_M105_S13.wav	105	C57BL/6	13	14	599.925024	0.38671499
USV_HOS_M105_S14.wav	105	C57BL/6	14	14	600.744224	0.570958465
USV_HOS_M106_S1.wav	106	C57BL/6	1	14	602.546464	3.833729243
USV_HOS_M106_S2.wav	106	C57BL/6	2	14	605.790496	1.223195156
USV_HOS_M106_S3.wav	106	C57BL/6	3	14	605.167904	1.455794325
USV_HOS_M106_S4.wav	106	C57BL/6	4	14	600.514848	1.005803607
USV_HOS_M106_S5.wav	106	C57BL/6	5	14	599.957792	1.200084422
USV_HOS_M106_S6.wav	106	C57BL/6	6	14	600.121632	2.639464928
USV_HOS_M106_S7.wav	106	C57BL/6	7	14	600.252704	0.644728458
USV_HOS_M106_S8.wav	106	C57BL/6	8	14	600.776992	1.007029244
USV_HOS_M106_S9.wav	106	C57BL/6	9	14	600.219936	1.847656057
USV_HOS_M106_S10.wav	106	C57BL/6	10	14	605.462816	0.191588975
USV_HOS_M106_S11.wav	106	C57BL/6	11	14	612.344096	0.200867455
USV_HOS_M106_S12.wav	106	C57BL/6	12	14	600.252704	0.728026708
USV_HOS_M106_S13.wav	106	C57BL/6	13	14	599.892256	0.383402182
USV_HOS_M106_S14.wav	106	C57BL/6	14	14	621.814048	0.262136246
USV_HOS_M201_S1.wav	201	C57BL/6	1	2	318.284064	3.053875798
USV_HOS_M201_S2.wav	201	C57BL/6	2	2	303.964448	1.723885815

USV_HOS_M202_S1.wav	202	C57BL/6	1	2	311.271712	3.79732547
USV_HOS_M202_S2.wav	202	C57BL/6	2	2	338.403616	1.858727183
USV_HOS_M203_S1.wav	203	C57BL/6	1	2	319.299872	2.881304005
USV_HOS_M203_S2.wav	203	C57BL/6	2	2	310.1576	2.466488005
USV_HOS_M204_S1.wav	204	C57BL/6	1	2	305.045792	1.589925227
USV_HOS_M204_S2.wav	204	C57BL/6	2	2	325.951776	0.460190774
USV_HOS_M205_S1.wav	205	C57BL/6	1	2	316.21968	2.042883605
USV_HOS_M205_S2.wav	205	C57BL/6	2	2	303.735072	1.106227206
USV_HOS_M206_S1.wav	206	C57BL/6	1	3	300.3272	2.663761392
USV_HOS_M206_S2.wav	206	C57BL/6	2	3	334.143776	2.424106203
USV_HOS_M206_S3.wav	206	C57BL/6	3	3	321.200416	1.509960685
USV_HOS_M207_S1.wav	207	C57BL/6	1	2	307.503392	2.43574549
USV_HOS_M207_S2.wav	207	C57BL/6	2	2	300.785952	1.858464453
USV_HOS_M208_S1.wav	208	C57BL/6	1	3	334.668064	2.497997538
USV_HOS_M208_S2.wav	208	C57BL/6	2	3	297.8696	1.252225806
USV_HOS_M208_S3.wav	208	C57BL/6	3	3	288.301344	1.845291432
USV_HOS_M209_S1.wav	209	C57BL/6	1	3	334.438688	3.531290017
USV_HOS_M209_S2.wav	209	C57BL/6	2	3	319.791392	3.261501173
USV_HOS_M209_S3.wav	209	C57BL/6	3	3	303.079712	3.35885234
USV_HOS_M210_S1.wav	210	C57BL/6	1	4	302.522656	3.649313458
USV_HOS_M210_S2.wav	210	C57BL/6	2	4	322.412832	1.271661545
USV_HOS_M210_S3.wav	210	C57BL/6	3	4	316.121376	1.755654765
USV_HOS_M210_S4.wav	210	C57BL/6	4	4	299.83568	0.280153449
USV_HOS_M301_S1.wav	301	C57BL/6	1	8	999.76016	0.756181363
USV_HOS_M301_S2.wav	301	C57BL/6	2	8	645.669152	0.859573356
USV_HOS_M301_S3.wav	301	C57BL/6	3	8	919.019808	1.121847419
USV_HOS_M301_S4.wav	301	C57BL/6	4	8	284.696864	1.447153278
USV_HOS_M301_S5.wav	301	C57BL/6	5	8	838.476064	0.025045438
USV_HOS_M301_S6.wav	301	C57BL/6	6	8	303.669536	3.526860198
USV_HOS_M301_S7.wav	301	C57BL/6	7	8	222.535968	1.307653781
USV_HOS_M301_S8.wav	301	C57BL/6	8	8	618.209568	0.079261148
USV_HOS_M303_S1.wav	303	C57BL/6	1	4	1467.261216	1.085014708
USV_HOS_M303_S2.wav	303	C57BL/6	2	4	929.997088	1.273122266
USV_HOS_M303_S3.wav	303	C57BL/6	3	4	1257.709856	1.552822371
USV_HOS_M303_S4.wav	303	C57BL/6	4	4	1222.09104	1.202856376
USV_HOS_M304_S1.wav	304	C57BL/6	1	4	1073.946912	1.818525645
USV_HOS_M304_S2.wav	304	C57BL/6	2	4	1213.669664	0.733313212
USV_HOS_M304_S3.wav	304	C57BL/6	3	4	994.484512	0.934152306
USV_HOS_M304_S4.wav	304	C57BL/6	4	4	1039.147296	1.246214088
USV_HOS_M305_S1.wav	305	C57BL/6	1	2	347.045888	2.091942377
USV_HOS_M305_S2.wav	305	C57BL/6	2	2	600.080384	3.20290423
USV_HOS_M306_S1.wav	306	C57BL/6	1	3	351.797248	2.797065655
USV_HOS_M306_S2.wav	306	C57BL/6	2	3	518.651904	3.420405837

USV_HOS_M306_S3.wav	306	C57BL/6	3	3	215.515136	0.890888703
USV_HOS_M307_S1.wav	307	C57BL/6	1	2	959.979808	1.667743412
USV_HOS_M307_S2.wav	307	C57BL/6	2	2	1387.24176	0.970991531
USV_HOS_M308_S1.wav	308	C57BL/6	1	2	1015.882016	1.555298721
USV_HOS_M308_S2.wav	308	C57BL/6	2	2	1015.292192	0.451101667
USV_HOS_M309_S1.wav	309	C57BL/6	1	1	1039.868192	1.558851413
USV_HOS_M310_S1.wav	310	C57BL/6	1	2	1157.636384	0.582220816
USV_HOS_M310_S2.wav	310	C57BL/6	2	2	1285.595424	1.434354826
USV_HOS_M401_S1.wav	401	C57BL/6	1	1	310.018048	3.590113567
USV_HOS_M402_S1.wav	402	C57BL/6	1	1	310.378496	2.619382497
USV_HOS_M403_S1.wav	403	C57BL/6	1	1	310.64064	2.974498122
USV_HOS_M404_S1.wav	404	C57BL/6	1	1	311.558144	2.484287492
USV_HOS_M405_S1.wav	405	C57BL/6	1	1	310.80448	3.88990532
USV_HOS_M406_S1.wav	406	C57BL/6	1	1	310.345728	3.215768448
USV_HOS_M407_S1.wav	407	C57BL/6	1	1	313.524224	2.605859253
USV_HOS_M501_S1.wav	501	C57BL/6	1	1	302.645248	0.888829419
USV_HOS_M502_S1.wav	502	C57BL/6	1	1	300.15488	2.155553827
USV_HOS_M503_S1.wav	503	C57BL/6	1	1	300.285952	3.147000363
USV_HOS_M504_S1.wav	504	C57BL/6	1	1	301.13792	2.646627832
USV_HOS_M601_S1.wav	601	C57BL/6	1	5	1200.422912	1.998462355
USV_HOS_M601_S2.wav	601	C57BL/6	2	5	1200.848896	1.681310618
USV_HOS_M601_S3.wav	601	C57BL/6	3	5	1184.333824	0.682240078
USV_HOS_M601_S4.wav	601	C57BL/6	4	5	933.298176	0.076074294
USV_HOS_M601_S5.wav	601	C57BL/6	5	5	1080.950784	0.614274035
USV_HOS_M602_S1.wav	602	C57BL/6	1	6	967.213056	1.637695015
USV_HOS_M602_S2.wav	602	C57BL/6	2	6	1181.974528	1.768227615
USV_HOS_M602_S3.wav	602	C57BL/6	3	6	990.24896	1.644031012
USV_HOS_M602_S4.wav	602	C57BL/6	4	6	1223.983104	0.304742769
USV_HOS_M602_S5.wav	602	C57BL/6	5	6	1041.563648	1.323970938
USV_HOS_M602_S6.wav	602	C57BL/6	6	6	1789.722624	0.143597671
USV_HOS_M603_S1.wav	603	C57BL/6	1	17	900.431872	0.034427924
USV_HOS_M603_S2.wav	603	C57BL/6	2	17	905.216	0.152449802
USV_HOS_M603_S3.wav	603	C57BL/6	3	17	900.333568	0.046649377
USV_HOS_M603_S4.wav	603	C57BL/6	4	17	1008.074752	0.144830529
USV_HOS_M603_S5.wav	603	C57BL/6	5	17	900.988928	0.095450674
USV_HOS_M603_S6.wav	603	C57BL/6	6	17	1200.226304	1.933801977
USV_HOS_M603_S7.wav	603	C57BL/6	7	17	1200.259072	1.394698894
USV_HOS_M603_S8.wav	603	C57BL/6	8	17	1200.750592	1.111804574
USV_HOS_M603_S9.wav	603	C57BL/6	9	17	1202.126848	0.552354355
USV_HOS_M603_S10.wav	603	C57BL/6	10	17	960.167936	0.393681132
USV_HOS_M603_S11.wav	603	C57BL/6	11	17	960.233472	0.264519002
USV_HOS_M603_S12.wav	603	C57BL/6	12	17	300.023808	2.139830183
USV_HOS_M603_S13.wav	603	C57BL/6	13	17	300.023808	0.499960323

USV_HOS_M603_S14.wav	603	C57BL/6	14	17	300.023808	0.263312437
USV_HOS_M603_S15.wav	603	C57BL/6	15	17	300.023808	0.999920646
USV_HOS_M603_S16.wav	603	C57BL/6	16	17	1540.620288	0.164219569
USV_HOS_M603_S17.wav	603	C57BL/6	17	17	1721.46688	0.353768061
USV_HOS_M604_S1.wav	604	C57BL/6	1	5	1219.592192	2.54019337
USV_HOS_M604_S2.wav	604	C57BL/6	2	5	1222.017024	1.96642105
USV_HOS_M604_S3.wav	604	C57BL/6	3	5	1149.698048	1.656087008
USV_HOS_M604_S4.wav	604	C57BL/6	4	5	1201.831936	0.321176355
USV_HOS_M604_S5.wav	604	C57BL/6	5	5	1200.45568	1.536083365
USV_HOS_M605_S1.wav	605	C57BL/6	1	6	915.832832	1.010009652
USV_HOS_M605_S2.wav	605	C57BL/6	2	6	1201.143808	1.173048548
USV_HOS_M605_S3.wav	605	C57BL/6	3	6	1221.623808	1.088714866
USV_HOS_M605_S4.wav	605	C57BL/6	4	6	922.681344	0.34789909
USV_HOS_M605_S5.wav	605	C57BL/6	5	6	1200.586752	1.533416887
USV_HOS_M605_S6.wav	605	C57BL/6	6	6	1520.10752	0.679557194
USV_HOS_M606_S1.wav	606	C57BL/6	1	6	925.499392	0.72609448
USV_HOS_M606_S2.wav	606	C57BL/6	2	6	1024.393216	0.495903323
USV_HOS_M606_S3.wav	606	C57BL/6	3	6	1000.341504	0.794728597
USV_HOS_M606_S4.wav	606	C57BL/6	4	6	1215.85664	1.034661455
USV_HOS_M606_S5.wav	606	C57BL/6	5	6	988.151808	1.432978201
USV_HOS_M606_S6.wav	606	C57BL/6	6	6	1281.490944	1.509959949
USV_HOS_M607_S1.wav	607	C57BL/6	1	7	924.22144	0.710868599
USV_HOS_M607_S2.wav	607	C57BL/6	2	7	990.57664	0.629936115
USV_HOS_M607_S3.wav	607	C57BL/6	3	7	1000.40704	0.096960533
USV_HOS_M607_S4.wav	607	C57BL/6	4	7	836.501504	0.130304607
USV_HOS_M607_S5.wav	607	C57BL/6	5	7	1200.29184	1.417155348
USV_HOS_M607_S6.wav	607	C57BL/6	6	7	1576.46848	0.145261388
USV_HOS_M607_S7.wav	607	C57BL/6	7	7	931.987456	0.438847108
USV_HOS_M608_S1.wav	608	C57BL/6	1	10	940.802048	0.707906622
USV_HOS_M608_S2.wav	608	C57BL/6	2	10	981.532672	0.091693331
USV_HOS_M608_S3.wav	608	C57BL/6	3	10	918.126592	0.033764407
USV_HOS_M608_S4.wav	608	C57BL/6	4	10	1045.037056	0.512900473
USV_HOS_M608_S5.wav	608	C57BL/6	5	10	300.023808	4.053011687
USV_HOS_M608_S6.wav	608	C57BL/6	6	10	300.023808	1.849853195
USV_HOS_M608_S7.wav	608	C57BL/6	7	10	300.023808	1.939846053
USV_HOS_M608_S8.wav	608	C57BL/6	8	10	300.023808	1.763193407
USV_HOS_M608_S9.wav	608	C57BL/6	9	10	1516.306432	0.335024629
USV_HOS_M608_S10.wav	608	C57BL/6	10	10	1602.715648	0.183438653
USV_HOS_M609_S1.wav	609	C57BL/6	1	11	917.602304	0.030514309
USV_HOS_M609_S2.wav	609	C57BL/6	2	11	960.167936	0.051032739
USV_HOS_M609_S3.wav	609	C57BL/6	3	11	1046.478848	0.017200539
USV_HOS_M609_S4.wav	609	C57BL/6	4	11	985.759744	0.048693407
USV_HOS_M609_S5.wav	609	C57BL/6	5	11	960.1024	0.449952005

USV_HOS_M609_S6.wav	609	C57BL/6	6	11	1201.7664	0.389426764
USV_HOS_M609_S7.wav	609	C57BL/6	7	11	1180.237824	0.39398839
USV_HOS_M609_S8.wav	609	C57BL/6	8	11	970.26048	0.029888881
USV_HOS_M609_S9.wav	609	C57BL/6	9	11	660.2752	0.616409643
USV_HOS_M609_S10.wav	609	C57BL/6	10	11	984.84224	0.324925137
USV_HOS_M609_S11.wav	609	C57BL/6	11	11	1420.066816	2.199896487
USV_HOS_M610_S1.wav	610	C57BL/6	1	6	1400.143872	2.203345
USV_HOS_M610_S2.wav	610	C57BL/6	2	6	1140.293632	1.765334773
USV_HOS_M610_S3.wav	610	C57BL/6	3	6	1260.322816	1.770974842
USV_HOS_M610_S4.wav	610	C57BL/6	4	6	1269.334016	0.144170091
USV_HOS_M610_S5.wav	610	C57BL/6	5	6	1199.833088	2.086956948
USV_HOS_M610_S6.wav	610	C57BL/6	6	6	1367.605248	1.589639995
USV_HOS_M611_S1.wav	611	C57BL/6	1	5	1090.256896	2.098587965
USV_HOS_M611_S2.wav	611	C57BL/6	2	5	1140.752384	2.112640775
USV_HOS_M611_S3.wav	611	C57BL/6	3	5	1359.937536	2.153040065
USV_HOS_M611_S4.wav	611	C57BL/6	4	5	1209.9584	2.012465883
USV_HOS_M611_S5.wav	611	C57BL/6	5	5	1210.253312	2.70273997
USV_HOS_M612_S1.wav	612	C57BL/6	1	6	905.084928	1.210936086
USV_HOS_M612_S2.wav	612	C57BL/6	2	6	1199.702016	0.877717955
USV_HOS_M612_S3.wav	612	C57BL/6	3	6	1140.007036	2.360511747
USV_HOS_M612_S4.wav	612	C57BL/6	4	6	946.372608	0.177519931
USV_HOS_M612_S5.wav	612	C57BL/6	5	6	1210.253312	1.541825981
USV_HOS_M612_S6.wav	612	C57BL/6	6	6	1252.032512	1.184474034
USV_HOS_M613_S1.wav	613	C57BL/6	1	9	1377.370112	0.278066147
USV_HOS_M613_S2.wav	613	C57BL/6	2	9	1070.137344	0.822324354
USV_HOS_M613_S3.wav	613	C57BL/6	3	9	1106.08384	0.019889993
USV_HOS_M613_S4.wav	613	C57BL/6	4	9	1160.052736	1.649924991
USV_HOS_M613_S5.wav	613	C57BL/6	5	9	300.023808	4.509642115
USV_HOS_M613_S6.wav	613	C57BL/6	6	9	300.023808	2.54646458
USV_HOS_M613_S7.wav	613	C57BL/6	7	9	300.023808	2.616459025
USV_HOS_M613_S8.wav	613	C57BL/6	8	9	300.023808	1.38655663
USV_HOS_M613_S9.wav	613	C57BL/6	9	9	1586.46272	0.072488309
USV_HOS_M614_S1.wav	614	C57BL/6	1	11	662.700032	0.04526935
USV_HOS_M614_S2.wav	614	C57BL/6	2	11	960.331776	1.094413437
USV_HOS_M614_S3.wav	614	C57BL/6	3	11	1320.22272	2.047381823
USV_HOS_M614_S4.wav	614	C57BL/6	4	11	1127.514112	0.070065642
USV_HOS_M614_S5.wav	614	C57BL/6	5	11	1200.357376	1.797797925
USV_HOS_M614_S6.wav	614	C57BL/6	6	11	300.023808	4.602968042
USV_HOS_M614_S7.wav	614	C57BL/6	7	11	300.023808	2.903102943
USV_HOS_M614_S8.wav	614	C57BL/6	8	11	300.023808	2.419807963
USV_HOS_M614_S9.wav	614	C57BL/6	9	11	299.139072	1.397343374
USV_HOS_M614_S10.wav	614	C57BL/6	10	11	1552.023552	0.465199126
USV_HOS_M614_S11.wav	614	C57BL/6	11	11	957.022208	0.082547719

USV_HOS_M615_S1.wav	615	C57BL/6	1	5	1240.072192	2.068427965
USV_HOS_M615_S2.wav	615	C57BL/6	2	5	1220.739072	1.281191072
USV_HOS_M615_S3.wav	615	C57BL/6	3	5	1320.22272	3.288081575
USV_HOS_M615_S4.wav	615	C57BL/6	4	5	1220.575232	2.069516023
USV_HOS_M615_S5.wav	615	C57BL/6	5	5	880.082944	0.548811908
USV_HOS_M616_S1.wav	616	C57BL/6	1	8	1000.2432	0.288929732
USV_HOS_M616_S2.wav	616	C57BL/6	2	8	300.023808	2.759780983
USV_HOS_M616_S3.wav	616	C57BL/6	3	8	300.023808	0.946591545
USV_HOS_M616_S4.wav	616	C57BL/6	4	8	300.023808	0.063328308
USV_HOS_M616_S5.wav	616	C57BL/6	5	8	300.023808	1.133243399
USV_HOS_M616_S6.wav	616	C57BL/6	6	8	137.428992	0.109147275
USV_HOS_M616_S7.wav	616	C57BL/6	7	8	1506.574336	0.686989002
USV_HOS_M616_S8.wav	616	C57BL/6	8	8	1502.150656	0.401424449
USV_HOS_M617_S1.wav	617	C57BL/6	1	9	1200.29184	0.266601829
USV_HOS_M617_S2.wav	617	C57BL/6	2	9	300.023808	4.9496072
USV_HOS_M617_S3.wav	617	C57BL/6	3	9	300.023808	4.349654812
USV_HOS_M617_S4.wav	617	C57BL/6	4	9	300.023808	2.963098182
USV_HOS_M617_S5.wav	617	C57BL/6	5	9	300.023808	1.389889698
USV_HOS_M617_S6.wav	617	C57BL/6	6	9	200.114176	1.87393021
USV_HOS_M617_S7.wav	617	C57BL/6	7	9	1502.052352	0.265636547
USV_HOS_M617_S8.wav	617	C57BL/6	8	9	1439.3344	0.142426944
USV_HOS_M617_S9.wav	617	C57BL/6	9	9	1260.683264	0.277627228
USV_HOS_M705_S1.wav	705	C57BL/6	1	2	1559.691264	1.143816114
USV_HOS_M705_S2.wav	705	C57BL/6	2	2	1274.380288	1.845602935
USV_HOS_M706_S1.wav	706	C57BL/6	1	1	1200.095232	1.951511795
USV_HOS_M707_S1.wav	707	C57BL/6	1	2	1211.138048	1.482902798
USV_HOS_M707_S2.wav	707	C57BL/6	2	2	1159.888896	0.592298109
USV_HOS_M711_S1.wav	711	C57BL/6	1	2	1704.067072	3.043894272
USV_HOS_M711_S2.wav	711	C57BL/6	2	2	1259.896832	3.580451895
USV_HOS_M712_S1.wav	712	C57BL/6	1	2	1217.560576	2.156771542
USV_HOS_M712_S2.wav	712	C57BL/6	2	2	1294.270464	2.434576147
USV_HOS_M733_S1.wav	733	C57BL/6	1	2	864.91136	1.855681488
USV_HOS_M733_S2.wav	733	C57BL/6	2	2	640.909312	1.148368398
USV_HOS_M735_S1.wav	735	C57BL/6	1	2	1453.457408	2.218159253
USV_HOS_M735_S2.wav	735	C57BL/6	2	2	1200.011776	1.964980717
USV_HOS_M825_S1.wav	825	C57BL/6	1	1	1206.943744	0.849252507
USV_HOS_M827_S1.wav	827	C57BL/6	1	1	960.9216	1.062521646
USV_HOS_M838_S1.wav	838	C57BL/6	1	1	721.584128	2.556874422
USV_HOS_M843_S1.wav	843	C57BL/6	1	2	1277.362176	2.115296703
USV_HOS_M843_S2.wav	843	C57BL/6	2	2	1404.239872	0.542642333
USV_HOS_M844_S1.wav	844	C57BL/6	1	2	1200.160768	1.877248498
USV_HOS_M844_S2.wav	844	C57BL/6	2	2	1496.776704	0.722218616
USV_HOS_M845_S1.wav	845	C57BL/6	1	1	635.731968	1.672088323

USV_HOS_M847_S1.wav	847	C57BL/6	1	1	645.20192	2.43489666
USV_HOS_M848_S1.wav	848	C57BL/6	1	1	634.38848	4.03380591
USV_HOS_M849_S1.wav	849	C57BL/6	1	1	630.390784	0.155459126
USV_HOS_M850_S1.wav	850	C57BL/6	1	1	628.850688	2.216742427
USV_HOS_M853_S1.wav	853	C57BL/6	1	1	633.962496	1.750892217
USV_HOS_M854_S1.wav	854	C57BL/6	1	1	646.283264	0.029398874
USV_HOS_M855_S1.wav	855	C57BL/6	1	1	634.159104	0.018922696
USV_HOS_M860_S1.wav	860	C57BL/6	1	1	636.551168	0.997563168
USV_HOS_M862_S1.wav	862	C57BL/6	1	1	637.00992	2.309226205
USV_HOS_M863_S1.wav	863	C57BL/6	1	1	614.07232	2.01279224
USV_HOS_M864_S1.wav	864	C57BL/6	1	1	637.796352	0.498591751
USV_HOS_M865_S1.wav	865	C57BL/6	1	1	614.203392	2.445444
USV_HOS_M866_S1.wav	866	C57BL/6	1	1	634.322944	0.189178085
USV_HOS_M868_S1.wav	868	C57BL/6	1	1	674.299904	0.704434328
USV_HOS_M869_S1.wav	869	C57BL/6	1	1	885.293056	2.477145828
USV_HOS_M870_S1.wav	870	C57BL/6	1	1	659.652608	3.348732307
USV_HOS_M871_S1.wav	871	C57BL/6	1	1	613.122048	0.79103337
USV_HOS_M876_S1.wav	876	C57BL/6	1	1	622.75584	0.841421254
USV_HOS_M879_S1.wav	879	C57BL/6	1	1	605.126656	0.755213798
USV_HOS_M880_S1.wav	880	C57BL/6	1	1	610.828288	1.67641221
USV_HOS_M881_S1.wav	881	C57BL/6	1	1	614.465536	0.172507641
USV_HOS_M882_S1.wav	882	C57BL/6	1	2	622.526464	1.786269443
USV_HOS_M882_S2.wav	882	C57BL/6	2	2	1020.526592	0.367457353
USV_HOS_M883_S1.wav	883	C57BL/6	1	2	655.261696	3.894627163
USV_HOS_M883_S2.wav	883	C57BL/6	2	2	974.979072	1.138485976
USV_HOS_M884_S1.wav	884	C57BL/6	1	1	625.573888	3.131524568
USV_HOS_M885_S1.wav	885	C57BL/6	1	2	621.28128	1.490468214
USV_HOS_M885_S2.wav	885	C57BL/6	2	2	600.96514	1.054969678
USV_HOS_M886_S1.wav	886	C57BL/6	1	3	620.62592	2.67794165
USV_HOS_M886_S2.wav	886	C57BL/6	2	3	674.594816	0.410616852
USV_HOS_M886_S3.wav	886	C57BL/6	3	3	1378.516992	0.27928564
USV_HOS_M888_S1.wav	888	C57BL/6	1	1	631.144448	1.601852006
USV_HOS_M889_S1.wav	889	C57BL/6	1	4	634.814464	1.934423473
USV_HOS_M889_S2.wav	889	C57BL/6	2	4	301.006848	0.073088038
USV_HOS_M889_S3.wav	889	C57BL/6	3	4	440.40192	0.869660151
USV_HOS_M889_S4.wav	889	C57BL/6	4	4	611.024896	0.58590084
USV_HOS_M890_S1.wav	890	C57BL/6	1	2	659.554304	3.737372322
USV_HOS_M890_S2.wav	890	C57BL/6	2	2	435.355648	0.92338299
USV_HOS_M891_S1.wav	891	C57BL/6	1	1	631.11168	1.56073803
USV_HOS_M892_S1.wav	892	C57BL/6	1	1	610.074624	0.986764531
USV_HOS_M893_S1.wav	893	C57BL/6	1	1	771.063808	2.940093902
USV_HOS_M894_S1.wav	894	C57BL/6	1	1	611.483648	1.412956835
USV_HOS_M895_S1.wav	895	C57BL/6	1	1	588.152832	1.582921903

USV_HOS_M896_S1.wav	896	C57BL/6	1	1	586.416128	0.908910882
USV_HOS_M897_S1.wav	897	C57BL/6	1	1	591.855616	1.355060218
USV_HOS_M898_S1.wav	898	C57BL/6	1	1	655.818752	0.385777319
USV_HOS_M899_S1.wav	899	C57BL/6	1	1	604.24192	0.077783415
USV_HOS_M901_S1.wav	901	C57BL/6	1	1	543.195136	0.292712489
USV_HOS_M902_S1.wav	902	C57BL/6	1	1	607.256576	1.719207402
1 - cmmz3xnho05oleqckafhuq507spznl0r	SCC_3BB89473	B6D2F1/J			300.0272	1.293216082
2 - 0k4zqzdznjwwkwtomv9i9ya0bla8c1l0	SCC_3BB89473	B6D2F1/J			300.0272	1.396540047
3 - tl709bw991e3242ttzrxyg6gr6efbn8f	SCC_3BB89473	B6D2F1/J			300.0272	2.51643851
15 - r5ltaktv55m3s0pmh5b1y3wjbtvnkigo	SCC_3BB89482	B6D2F1/J			300.0272	2.139805992
16 - wu1ndwzuvw34f365rp9lwanefznxyto8	SCC_3BB89482	B6D2F1/J			300.0272	2.406448482
17 - rgitzuyoj3jcn33nh6xjwnscr9p7iw6b	SCC_3BB89482	B6D2F1/J			300.0272	3.10305199
29 - wmebenkgk8llqvwehctwf8xsqh2dvbaw	SCC_3BB89487	B6D2F1/J			300.023808	2.00650743
30 - c4v05vrsm2gyuih0k19mi77sbiqn8v8o	SCC_3BB89487	B6D2F1/J			300.0272	2.679757035
31 - og2vz0iej6kqzdn9bkd12y9rt7jspm0h	SCC_3BB89487	B6D2F1/J			300.0272	2.596431257
43 - thgk6bh0z3wv717k64nxm99ekwsds9ia	SCC_3BB89488	B6D2F1/J			300.0272	1.923158967
44 - u7gh89uudbki5iacojzkmr4ke31gqcgu	SCC_3BB89488	B6D2F1/J			300.0272	1.266551833
45 - l20rz803d44qnn7swxcdwxzwnlybkffc	SCC_3BB89488	B6D2F1/J			300.0272	0.5566162
57 - 5e55dxug9f2vh1vpht2qpe3h0hgy1dqj	SCC_3BB8948F	B6D2F1/J			300.0272	0.869921127
58 - fydj8jbk0jlhrqxyj0rgd8hdask60r82	SCC_3BB8948F	B6D2F1/J			300.0272	1.886495625
59 - bkbjhbjcerrbvd541wdbphh3h3sacgha	SCC_3BB8948F	B6D2F1/J			300.0272	0.256643398
71 - edtkmx90m87w85nc3u0kw02ylfm8f32x	SCC_3BB8949D	B6D2F1/J			300.0272	1.556525542
72 - syozldpm9wdvg4fnunzpaawrjkxpeay4	SCC_3BB8949D	B6D2F1/J			300.0272	1.81316894

73 - fa5ipfrwizrb3xpeiayyh6z63yg3mu5z	SCC_3BB8949D	B6D2F1/J	300.0272	1.683180725
85 - uc87ghup5ft5siazk0pbk3czdm2dtl4s	SCC_3BB894AD	B6D2F1/J	300.0272	2.073145368
86 - lu9pf9bp0g4t6xg8ufwrkwpjsbajagqp	SCC_3BB894AD	B6D2F1/J	300.0272	1.266551833
87 - y2qz9rgf311qvlg0dvltcnixtq0rqqvu	SCC_3BB894AD	B6D2F1/J	300.0272	1.826501063
100 - pq9bp4d5yl7iwsy4fxv4w1hon2jra0xy	SCC_3BB894B6	B6D2F1/J	300.0272	2.10980871
101 - jyopyk71eoldm9zbo0mwoqlogxgwjw21	SCC_3BB894B6	B6D2F1/J	300.0272	3.789656405
99 - xamdb7kt8znhscr7x5x1hn467ahhkm7a	SCC_3BB894B6	B6D2F1/J	300.0272	1.256552739
113 - 87istbrqh8fcdlkfa8gdgwkndjy8c7qt	SCC_3BB894B7	B6D2F1/J	300.0272	1.556525542
114 - 1xdcs6eoen3yfexyo3kc11iv4llknydz	SCC_3BB894B7	B6D2F1/J	300.0272	1.863164407
115 - zmi4wlgx9rwa1sa3cewt31wwhrcxbujn	SCC_3BB894B7	B6D2F1/J	300.0272	1.706511943
127 - vu6a8xsoatlbckrcjalubgvj0q0cxml6	SCC_3BB894B9	B6D2F1/J	300.0272	3.669667283
128 - sm8jyvebt5no7tt0mrs74z7v6mpb1h0g	SCC_3BB894B9	B6D2F1/J	300.0272	2.563100945
129 - jnpihx07wsqhmh1lyw4zj896rrgtif4e	SCC_3BB894B9	B6D2F1/J	300.0272	0.266642491
141 - 82rh2ctdfbakrq1sv463ei83hcfv64ch	SCC_3BB894BC	B6D2F1/J	300.0272	1.95982231
142 - j7pwcq2zn3b1d45ftnrdjjynbpz06xh2	SCC_3BB894BC	B6D2F1/J	286.166336	3.599305265
143 - 2cs40chs8zjlx2vn6229rokwdnibt00d	SCC_3BB894BC	B6D2F1/J	300.023808	3.423061678
155 - 6a83nglngoetrrgptucw8ihtf182kabf	SCC_3BB894CE	B6D2F1/J	300.0272	3.096385928
156 - mw8c0eo6supphyosedz4wyp6ift5bncb	SCC_3BB894CE	B6D2F1/J	300.0272	3.673000315
157 - azyxo2bopr3efhh8o8fzdo6ka7jkn0fx	SCC_3BB894CE	B6D2F1/J	300.0272	3.649669097

SUPPLEMENTARY TABLE 1 | Mice and recording sessions in the USV database. The supplementary table that contains information about the mice and recordings sessions in the USV database. Each row stores data about a single session (session ID, length and syllable rate) and about the male mouse recorded in that session (mouse ID, strain and the total number of sessions).
