# Supplementary material for: Temporal structure of mouse courtship vocalizations facilitates syllable labeling
Source: Commun Biol. 2020 Jun 26;3:333. doi: 10.1038/s42003-020-1053-7 (PMC7320152; doi:10.1038/s42003-020-1053-7)
Supplement: Supplementary file 3 — Description of Additional Supplementary Files [file 42003_2020_1053_MOESM3_ESM.pdf]

Description of Additional Supplementary Files

File Name: **Supplementary Data 1**
Description: All data used to create figures and charts of the paper
