# Supplementary material for: Temporal structure of mouse courtship vocalizations facilitates syllable labeling
Source: Commun Biol. 2020 Jun 26;3:333. doi: 10.1038/s42003-020-1053-7 (PMC7320152; doi:10.1038/s42003-020-1053-7)
Supplement: Supplementary file 4 — Reporting Summary [file 42003_2020_1053_MOESM4_ESM.pdf]

nature research

Corresponding author(s): Michael London

Last updated by author(s): Feb 7, 2020

Reporting Summary

Nature Research wishes to improve the reproducibility of the work that we publish. This form provides structure for consistency and transparency in reporting. For further information on Nature Research policies, see Authors & Referees and the Editorial Policy Checklist.

Statistics

For all statistical analyses, confirm that the following items are present in the figure legend, table legend, main text, or Methods section.

n/a	Confirmed	
☐	☒	The exact sample size (*n*) for each experimental group/condition, given as a discrete number and unit of measurement
☐	☒	A statement on whether measurements were taken from distinct samples or whether the same sample was measured repeatedly
☐	☒	The statistical test(s) used AND whether they are one- or two-sided<br>*Only common tests should be described solely by name; describe more complex techniques in the Methods section.*
☒	☐	A description of all covariates tested
☒	☐	A description of any assumptions or corrections, such as tests of normality and adjustment for multiple comparisons
☐	☒	A full description of the statistical parameters including central tendency (e.g. means) or other basic estimates (e.g. regression coefficient) AND variation (e.g. standard deviation) or associated estimates of uncertainty (e.g. confidence intervals)
☐	☒	For null hypothesis testing, the test statistic (e.g. *F*, *t*, *r*) with confidence intervals, effect sizes, degrees of freedom and *P* value noted<br>*Give P values as exact values whenever suitable.*
☒	☐	For Bayesian analysis, information on the choice of priors and Markov chain Monte Carlo settings
☒	☐	For hierarchical and complex designs, identification of the appropriate level for tests and full reporting of outcomes
☐	☒	Estimates of effect sizes (e.g. Cohen's *d*, Pearson's *r*), indicating how they were calculated

Our web collection on statistics for biologists contains articles on many of the points above.

Software and code

Policy information about availability of computer code

Data collection	For ultrasonic vocalization recording we used an UltraSoundGate system (Avisoft bioacustic, Germany) composed of a CM16/CMPA ultrasound microphone, UltraSoundGate 116H computer interface, and USGH recorder software on a standard PC computer. Sampling frequency of 250 KHz and 16-bit recordings were used. For online monitoring we used simultaneous display of the spectrogram (256 points FFT).
Data analysis	* Python 2.7<br>* MATLAB 2018b<br>* USV parser:<br>After testing a few parsing tools, we noticed that they were not optimized to cope with the different types of noise that existed in the USV files. These different levels of noise in the recordings were a result of: varying cage sizes, cage acoustics, locations of the recording device and noise from the freely moving mice. Therefore, we developed a USV parser (written in Python; available online) that is robust to these types of noise. Figure S7 describes the flow of the parser. The parser receives as input one or more 'wav' USV files and returns the start and end time of each syllable in the file.<br>* Adaptation of existing algorithms<br>Source code for all three algorithms was available in MATLAB. We performed several adaptations to each algorithm in order to enable a fully-automated execution.<br>Mouse Song Analyzer v1.3 (Chabout et al., 2015)<br>The MSA algorithm includes a built-in syllable parser. In order to label the same syllables for all algorithms, we replaced the syllables detected by the MSA parser with those that were detected by our parsing algorithm (see above). We ran the MSA algorithm on all files and saw that there were files where more than 5% of the syllables were labelled as "unclassified". For those files, we re-ran the algorithm with lower and lower thresholds (default was 0.3, decrease steps were of 0.05 and minimum value was 0.15). We selected the first threshold with an "unclassified" rate lower than 5%. If there was no such threshold, we selected the threshold with the lowest unclassified rate. Nevertheless, manual examination of the remaining "unclassified" syllables showed still a significant amount of real USVs. The shorter "unclassified" syllables were "simple" and the longer ones tended to be "multiple". As a result, and in order to classify each one of the syllables to one of the four basic classes (simple, down, up, multiple), we gave the "unclassified" syllables one of two

labels: “simple” or “multiple”. We used the median duration of all syllables in the database (35.3 ms) and set the syllables with a shorter duration as “simple” and with a longer duration as “multiple”. In total there were 39,992 "unclassified" syllables of which 29,246 were classified as simple (19.2% of the total simple population) and 10,746 were classified as multiple (18.3% of total multiple). Next, to support an eight-class classification, we split each one of the four classes to two. This was done using the median duration of each class (simple: 27.6 ms, down: 48.1 ms, up: 50.7 ms and multiple: 96.3 ms). For example, “down” syllables that were shorter than 48.1 ms were classified as Down-short and “up” syllables longer than 50.7 ms were classified as Up-long.

VoICE (Burkett et al., 2015)
The VoICE algorithm is based on hierarchical clustering. Running the algorithm on all syllables in the database was not feasible because of computation constrains. Therefore, 4000 syllables from different files were selected and the algorithm was applied to them. VoICE includes a manual phase (originally used for comparison) that was skipped. The results of the automatic phase are centroids that were further used to classify all 346,632 syllables. This classification was done using the same similarity measure that was used in the other parts of the VoICE algorithm.

MUPET (Van Segbroeck et al., 2017)
MUPET uses a gammatone filter as part of the preprocessing. For the adapted version, we used 16 filters. As in the MSA algorithm, MUPET also contains a parsing phase. We loaded our syllable times instead of the built-in ones to maintain consistency. Like the case with VoICE, the MUPET algorithm was not able to run on the full database, therefore we applied it on 5000 syllables. Then, we used the resulting centroids and the MUPET distance measure to classify the rest of the syllables.

For manuscripts utilizing custom algorithms or software that are central to the research but not yet described in published literature, software must be made available to editors/reviewers. We strongly encourage code deposition in a community repository (e.g. GitHub). See the Nature Research guidelines for submitting code & software for further information.

Data

Policy information about availability of data

All manuscripts must include a data availability statement. This statement should provide the following information, where applicable:

- Accession codes, unique identifiers, or web links for publicly available datasets
- A list of figures that have associated raw data
- A description of any restrictions on data availability

The full set of files used in this study was uploaded to mouseTube and can be found using the group label “London Lab”. In addition, Supplementary Table 1 contains information about the recording sessions and the male mouse that was recorded during that session

Field-specific reporting

Please select the one below that is the best fit for your research. If you are not sure, read the appropriate sections before making your selection.

☒ Life sciences ☐ Behavioural & social sciences ☐ Ecological, evolutionary & environmental sciences

For a reference copy of the document with all sections, see nature.com/documents/nr-reporting-summary-flat.pdf

Life sciences study design

All studies must disclose on these points even when the disclosure is negative.

Sample size	Parsing 385 recording sessions resulted with 346,632 syllables grouped into 33,481 sequences.
Data exclusions	We have scrutinized our database for duplicates and low-quality USVs which resulted in discarding some of the data.
Replication	The entropy rates and SIS values were computed over 25 repetitions. In each repetition, 60% of the sequences in the database are used to construct the suffix tree which the values are calculated from. The mean value of the 25 repetitions is plotted as a dot and the error bars mark 2 standard deviations.
Randomization	See above.
Blinding	n/a

Reporting for specific materials, systems and methods

We require information from authors about some types of materials, experimental systems and methods used in many studies. Here, indicate whether each material, system or method listed is relevant to your study. If you are not sure if a list item applies to your research, read the appropriate section before selecting a response.

Materials & experimental systems

n/a	Involved in the study
☒	☐ Antibodies
☒	☐ Eukaryotic cell lines
☒	☐ Palaeontology
☐	☒ Animals and other organisms
☒	☐ Human research participants
☒	☐ Clinical data

Methods

n/a	Involved in the study
☒	☐ ChIP-seq
☒	☐ Flow cytometry
☒	☐ MRI-based neuroimaging

Animals and other organisms

Policy information about studies involving animals; ARRIVE guidelines recommended for reporting animal research

Laboratory animals	For the recordings performed in our lab, we used C57BL/6 male and female mice (8–12 weeks old). All mice were group-housed (3–4 per cage) and kept on a 12 h light (7 a.m.–7 p.m.)/dark cycle with ad libitum food and water.
Wild animals	The study did not involve wild animals.
Field-collected samples	The study did not involve samples collected from the field.
Ethics oversight	Experimental protocols were approved by the Hebrew University Animal Care and Use Committee and met guidelines of the National Institutes of Health guide for the Care and Use of Laboratory Animals (IACUC NS-16-14216-3).

Note that full information on the approval of the study protocol must also be provided in the manuscript.
